# Integrins Are the Necessary Links to Hypertrophic Growth in Cardiomyocytes

**DOI:** 10.1155/2011/521742

**Published:** 2011-02-21

**Authors:** Rebecca K. Harston, Dhandapani Kuppuswamy

**Affiliations:** ^1^Cardiology Division, Department of Medicine, Gazes Cardiac Research Institute, Medical University of South Carolina, Charleston, SC 29425-2221, USA; ^2^Advanced BioScience Laboratories, Inc., 5510 Nicholson Lane, Kensington, MD 20895, USA

## Abstract

To compensate for hemodynamic overload of the heart, an event which stretches the myocardium, growth and survival signaling are activated in cardiac muscle cells (cardiomyocytes). Integrins serve as the signaling receptors of cardiomyocytes responsible for mechanotransduction toward intracellular signaling. The main integrin heterodimers on the cardiomyocyte surface are *α*_5_ *β*_1_ and *α*_*v*_ *β*_3_, and elimination of either *β*_1_ or *β*_3_ integrins impedes pressure-induced hypertrophic signaling and leads to increased mortality. The growth signaling pathways downstream of *β*_1_ and *β*_3_ integrins are well characterized. However, new integrin pathways responsible for inhibiting apoptosis induced by hemodynamic overload are emerging. *β*_1_ and *β*_3_ integrins activate differential survival signaling, yet both integrins initiate survival signaling downstream of ubiquitination and the kinase pathway including phosphoinositol-3-kinase (PI3K)/Akt. Further characterization of these integrin-signaling mechanisms may lead to drug targets to prevent decompensation to heart failure.

## 1. Cardiac Hypertrophy

Cardiac hypertrophy begins as a compensatory response to hemodynamic overload, such as from increased pressure with hypertension. Several cell types are present in the ventricular wall that provide diverse contributions for optimal organ performance. Cardiomyocytes are the muscle cells in the heart which make up myofibrils that contract to pump blood to the body. Like skeletal muscle cells, cardiomyocytes are striated, but only cardiomyocytes have intercalated discs, which connect adjacent cells and help them simultaneously contract. While all cell types in the heart differentially respond to hemodynamic changes, the cardiomyocytes ultimately regulate cardiac performance through pump function and must change intracellular signaling pathways most substantially. 

Hypertrophy is defined by the increase in protein synthesis, cardiomyocyte size, and cytoskeletal remodeling, which are important to increase the ventricle wall thickness to normalize against the mechanical stress [1] and optimize contractility (reviewed in [2]). Cardiomyocytes are terminally differentiated and, therefore, have limited proliferative potential. Thus, cell growth and initiation of survival signaling are their means of increasing ventricle muscle mass. As cardiomyocytes reenter a growth phase not initiated since development, there is an immediate early gene induction of proto-oncogenes, and heat shock protein genes are activated first, followed by induction of a “fetal” gene program similar to developmental expression patterns. However, hypertrophic growth often results in heart failure by a poorly understood mechanism when the heart can no longer compensate for sustained pressure overload (PO). Hypertension is the leading cause for heart disease and stroke in the United States and thus remains an important area to study for a beneficial impact on human health.

## 2. Hypertrophic Growth by Integrin Activation

Hemodynamic overload of the heart instigates many stimuli received by cardiomyocytes for induction of growth, including neurohormonal changes, mobilization of growth factors, stretch of the myocardium, and extracellular matrix (ECM) remodeling. Many of these upstream events either utilize or synergize integrin receptors to initiate growth and survival signaling. Cardiomyocytes require tight regulatory processes to elicit signaling changes in response to hypertrophic stimuli. In addition, cytoskeletal dynamics must be able to change, but remain functional in order for each cardiomyocyte to enlarge, add sarcomeres, and extend their cell borders within the heart tissue [1]. Targeted protein synthesis, which is required for growth, is increased due to amplified protein synthesis rates and machinery [3, 4]. These changes, proceeding as part of the compensatory mechanism, normalize wall stress and optimize contractility without the ability to increase cell number [2]. 

Other receptors such as G protein-coupled receptors (GPCRs) and receptor tyrosine kinases (RTKs) are known to be activated by autocrine and paracrine factors released during mechanical stress and can function in concert with integrins to activate downstream kinase pathways such as phosphotidylinositol-3-kinase (PI3K). For instance, humoral factors released during hemodynamic overload activate *β*-adrenoreceptors and growth factor receptors which may elicit downstream signaling through synergistic integrins. GPCRs activate matrix metalloproteinases (MMPs), MMP-2, MMP-7, MMP-9, and a disintegrin and metalloproteinases (ADAMs), ADAM-12, and ADAM-17. MMPs cleave ECM proteins, resulting in activation of hypertrophic signaling and induction of fetal genes. One recent study showed angiotensin II-induced GPCRs induce MMP-7 which transcriptionally activates ADAM-12. This signaling axis is required for angiotensin-induced hypertrophic signaling [5].

However, mechanical stimulation alone also begets hypertrophic growth [6] through integrin activation [7]. In fact, simply stretching isolated cardiomyocytes results in immediate-early gene and fetal gene induction [8], proto-oncogene expression [9], and increased protein synthesis [10]. In a feline model of hemodynamic overload, simultaneous inhibition of *α*- and *β*-adrenoreceptors during pressure overload did not affect hypertrophic growth [6]. Therefore, growth can be directly mediated by mechanical stimulation of the myocardium, and integrins are the necessary receptors that physically sense and respond to stretch [11].

## 3. Integrin Heterodimers in the Heart

Integrins are a class of heterodimeric transmembrane proteins made up of the noncovalent interaction of *α* and *β* subunits. Extracellular domains function for adhesion and ligand recognition, and upon activation, short cytoplasmic domains of *β* subunits, which lack kinase function, physically connect to the cytoskeleton and recruit proteins for signaling [12]. The integrin subunits consist of at least 18 *α* subunits and 8 *β* subunits, which in various combinations make up 24 different discovered integrin heterodimers [12]. These heterodimers elicit varying functions and are expressed in a tissue-specific manner [11]. The main integrin heterodimers on the cardiomyocyte surface for activation during hypertrophy are *α*_5_ *β*_1_ and *α*_*v*_ *β*_3_. As physical links between the intracellular cytoskeleton and either the extracellular matrix or the adjacent cardiomyocyte, integrins are regulators of adhesion and mechanotransduction. Integrins are basally expressed in the intercalated disc [13] and costameres of cardiomyocytes [14, 15]. Intercalated discs are protein complexes at the Z lines of cardiomyocytes, providing sites of cell-cell interactions. Costameres are adjacent to the Z lines in cardiomyocytes and offer connection sites for cell-extracellular matrix interactions. Most integrin heterodimers bind all ECM proteins while the *α*_5_ *β*_1_ only binds to fibronectin [16]. The importance of integrins for mechanotransduction and/or adhesion through these interactions is evidenced by integrin upregulation in the intercalated disc upon damage to the myocardium to make new extracellular matrix contacts for intracellular signaling [17, 18]. Both *β*_3_ and *β*_1_ integrins have been identified at intercalated discs [17–20] and costameres [15, 21]. To mediate signaling, *β*_1_ integrin translocates to the intercalated disc in damaged areas of the myocardium after infarct [17] while *β*_3_ integrin is upregulated to the cell surface in RGD-stimulated adult cardiomyocytes [22]. Additionally, *α*_5_ *β*_1_ has been shown to be important for adherence of cardiomyocytes [11] and is necessary for growth in rat neonatal cardiomyocytes [23–25]. 

Prior to mechanical stimulation, integrins remain freely mobile singular heterodimers on the cell surface, meaning they do not have a constitutive connection to extracellular matrix proteins or cytoskeletal proteins. Integrins that respond to mechanical stretch of the myocardium, namely, *α*_5_ *β*_1_ and, mediate their signals by recognizing the specific amino acid sequence Arg-Gly-Asp (RGD) in ECM proteins, such as collagen, vitronectin, and fibronectin [16]. Although various integrin subunits have specificities to RGD motifs of various ECM proteins including fibronectin, vitronectin, and collagen, many of them have recognition motifs in the non-RGD motifs of ECM proteins, however the *α*_1_, *α*_5_, and *β*_3_ subunits have RGD specificity [12]. The *β*_1_ subunit is more promiscuous, and its role in binding to RGD is dependent on the associated *α* subunit [12]. For example, by pairing with *α*_5_ subunit, *β*_1_ integrin interacts primarily with the RGD motif of fibronectin. In intact myocardium, tortional stress induced in the tissue from increased blood pressure causes structural alterations of ECM proteins which results in exposure of their RGD motifs. These extracellular matrix proteins are also genetically upregulated and secreted with hypertrophic induction in the immediate early gene response of cardiomyocytes [22, 26, 27]. Concurrently, integrin activation at the cardiomyocyte cell surface also increases [19]. 

Since integrins are positioned to sense stretch of the myocardium, both the *β*_1_ and *β*_3_ integrins have roles in hypertrophic growth. During hypertrophic induction, several integrin subunits are upregulated and/or activated: *α*_1_, *α*_5_, *β*_1_, and *β*_3_ [19, 28], suggesting their role in promoting cardiac hypertrophy. Genetic ablation of either *β*_3_ or *β*_1_ integrins inhibits pressure-induced hypertrophic signaling, resulting in reduced cardiac output with increased mortality and heart failure [18, 20]. It is clear *β*_1_ integrin plays a role in *β*-adrenergic receptor-induced hypertrophy [23–25, 29–31]. In neonatal rat ventricular cardiomyocytes, overexpression of *β*_1_ integrin activates expression of the immediate early gene, atrial natriuretic factor (ANF). Conversely, blocking *β*_1_ integrin prevents ANF expression during *α*_1_-adrenergic stimulation [23]. A heterozygous conditional *β*_1_ knockout mouse subjected to isoproterenol exhibits less hypertrophic growth and more apoptosis (TUNEL-positive nuclei) compared to wild-type mice [29]. Additionally, *β*_1_ integrin is involved in another humoral hypertrophic pathway, as blocking antibodies for *β*_1_ integrin in a stretch model of adult rat cardiomyocytes reduced Ang II secretion [31]. However, expression of a truncated form of *β*_1_ integrin without the cytoplasmic domain in RGD-stimulated adult cardiomyocytes renders *β*_1_ integrin incapable of recruiting specific focal adhesion molecules, though focal adhesion complex (FAC) formation itself is not affected [19]. In the case of *β*_3_ integrin, our recent work demonstrates that FAC formation and subsequent downstream signaling in pressure-overloaded myocardium occurs through this integrin activation [20]. That is, transverse aortic restriction in *β*_3_ integrin knockout mice results in a deficiency of compensatory hypertrophic growth, evidenced by stunted hypertrophic growth, decreased ventricular function and geometry, and increased mortality with compromised survival signaling [20]. Furthermore, restriction of p70 ribosomal S6 kinase activation in cell culture is observed when inhibiting *β*_3_ integrin signaling but not with *β*_1_ integrin inhibition with specific blocking antibodies [32]. This observation indicates distinct effects of these two integrin subtypes. 

Upon integrin activation from pressure-induced hypertrophy, multiple downstream signaling cascades mediate protein synthesis, survival, and gene expression: (i) there is evidence both *β*_1_ and *β*_3_ integrins initiate downstream survival signaling utilizing PI3K/Akt and ubiquitination activation [30, 33, 34], (ii) mechanical stimulation of cardiomyocytes and pressure overload *in vivo* have been shown to initiate FAC formation [7, 22] and downstream signaling via Ras-ERK1/2 and Akt [35], (iii) *β*_3_ integrin activates caspase inhibiting molecules for survival signaling in smooth muscle cells [36], cardiomyocytes [20], and hypertrophic myocardium [32, 33], and (iv) it is well documented that hypertrophic induction of integrins results in the activation of mammalian target of rapamycin (mTOR) for increased protein synthesis and cell growth [32]. The survival signaling regulated by ubiquitination downstream of integrin activation is discussed in more detail below.

## 4. Integrins Link Mechanical and Biochemical Signals via Activation of Nonreceptor Tyrosine Kinases

**Figure 1:**
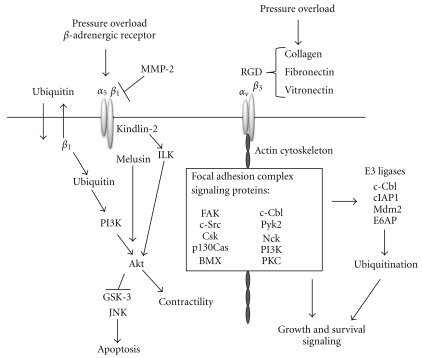
Integrin signaling in hypertrophic growth (see text for details).

Once activated after recognition of extracellular matrix proteins, integrins cluster on the cell surface, associate with cytoskeletal proteins, and recruit signaling molecules onto the actin cytoskeleton to form a complex of scaffolding proteins and kinases called a FAC. Since the cytoplasmic tails of integrins do not possess kinase function on their own, recruitment of kinase molecules into the FAC is required for initiation of intracellular signaling and downstream gene expression (Reviewed in [37]). In this way, integrins are a physical link between the extracellular matrix and the intracellular cytoskeleton and transduce mechanical signals into intracellular biochemical signals. The FAC is made up of nonreceptor tyrosine kinases (NTKs) [7, 22], such as FAK, Pyk2, cSrc, cSrc-kinase (Csk), c-Cbl and other Ser/Thr kinases, that is, PI3K [38] and PKC [39]. FAK is one of the first kinases recruited to the *β* integrin cytoplasmic tail upon stimulation [40]. After autophosphorylation of Tyr397-FAK, SH2 domain-containing proteins are recruited, which include cSrc [41], Nck [42], and PI3K [43]. With recruitment of protein kinases to this complex, intracellular kinase cascades are then activated for growth and survival signaling and gene expression (Figure 1). 

The FAK/Src complex mediates hypertrophic growth and survival signaling in the adult rat heart activated by mechanical stimulation from load/pressure [22, 43]. Evidence shows integrin activation of FAK requires Src while GPCR activation of FAK is Src-independent [44]. FAK phosphorylation and translocation in stretch-induced neonatal rat cardiomyocytes induces immediate-early gene expression and hypertrophic signaling. With 5–20% cyclic stretch, Tyr397-FAK is increased, and FAK translocates from around the nucleus to the myofilaments to activate downstream signaling, including expression of atrial natriuretic factor (ANF) [44]. Therefore, both recruitment and activation of NTKs are critical for the mediation of hypertrophic signaling.

## 5. *β*_1_ Integrin Signals through the *β*-Adrenergic Receptor

At intercalated discs and costameres, *β*_1_ integrin engages with several critical proteins (illustrated in Figure 1). The focal adhesion protein kindlin-2 is concentrated at intercalated discs and costameres [45]. Kindlin-2 has been proven an important molecule for integrin-mediated adhesion [46] and intercalated disc functionality [47] and has also been shown to activate the major survival kinase integrin-linked kinase (ILK), which binds the cytoplasmic tail of *β*_1_ integrins to transduce biomechanical signals received by the integrin into biochemical signaling within the cardiomyocyte. Since cardiac-specific knockout of ILK causes spontaneous cardiac failure while cardiac-specific knockouts of *β*_1_ integrin, melusin, or FAK require the stress of pressure overload to decompensate to cardiac failure, ILK likely has other signaling effectors to mediate several critical pathways [48, 49]. No investigation in cardiomyocytes of the activation of ILK by *β*_3_ integrin has been published to date. The activation of Akt by ILK has been shown to be necessary for cardiomyocyte contractility, and phosphorylation at S473 of Akt was abolished in *ilk^−/−^* hearts. The crucial role of ILK in normal cardiac function could be explained by its activation of Akt and because ILK transduces signals from not only mechanical stimulation but also growth factors and cytokines [49]. 

*β*_1_ integrins also interact with the muscle-specific protein melusin [15]. Genetic disruption of melusin does not affect humoral hypertrophic pathways elicited by angiotensin II or phenylephrine (PE), but melusin is required for pressure-induced hypertrophy [50]. *β*-adrenergic receptor stimulation in adult cardiomyocytes results in apoptosis downstream of both GSK3*β* and c-Jun N-terminal kinase (JNK) activation, which is mediated by the interference of *β*_1_ integrin with the matrix metalloproteinase-2 (MMP-2) [51]; in fact, blocking MMP-2 activation ablates myocardial remodeling and dysfunction during chronic pressure overload [52]. Similarly, disrupting the gene for melusin inhibits Akt-mediated GSK3*β* phosphorylation and subsequent inhibition during pressure overload [50] while overexpressing melusin inhibits pressure-overload induced apoptosis [53].

## 6. Ubiquitin-Mediated Survival Signaling Downstream of *β*_1_ Integrin

Both *β*_1_ integrin activation [30] and ubiquitin expression [34] can activate the PI3K/Akt pathway to inhibit apoptosis. Ubiquitin was recently found to be excreted by adult cardiomyocytes undergoing *β*-adrenergic stimulation by isoproterenol. The ubiquitin is also taken in by the cardiomyocytes and is required for the PI3K-dependent antiapoptotic pathways activated [34]. As described, stimulating *β*-adrenergic receptor in adult cardiomyocytes induces apoptosis, measured by terminal deoxynucleotidyl transferase-mediated nick end labelling (TUNEL) staining, by activating GSK3*β* for subsequent JNK activation [49]. Addition of ubiquitin to the media of cardiomyocytes undergoing *β*-adrenergic receptor inhibited apoptosis, JNK activation, and cytochrome c release; further PI3K activation was required [40], since PI3K activates Akt, which directly inhibits GSK3*β*. Though not fully investigated, these data indicate the possible link between *β*_1_ integrin and ubiquitin in antiapoptotic signaling.

## 7. Survival Signaling Downstream of *β*_3_ Integrin and Ubiquitination

In *β*_3_ integrin signaling, hypertrophic stimulation leads to FAC formation, ubiquitination of specific proteins, and NF*κ*B activation [20]. Synonymous with integrin-mediated signaling, molecules involved in the ubiquitin-mediated pathway are recruited to the cytoskeleton in order to elicit downstream signaling. *β*_3_ integrin upregulates ubiquitination near intercalated discs of cardiomyocytes within the first week of pressure overload [20]; this is the time period of tyrosine kinase activation downstream of FAC formation [7, 19, 22]. Through specific ubiquitination of the inhibitor of *κ*B (I*κ*B) during pressure overload of the heart, NF*κ*B is activated and transcribes the cellular inhibitor of apoptosis protein 1 (cIAP1) [20]. Accordingly, NF*κ*B is also regulated by FAK activation during mechanical stimulation of cardiomyocytes [54]. NF*κ*B functions as a survival transcription factor known to be required for hypertrophic growth [55], and one of its transcripts is the cellular inhibitor of apoptosis (cIAP), an E3 ligase that causes ubiquitin-mediated removal of caspases [56]. E3 ligases are the enzymes responsible for recognizing and tagging specific proteins with ubiquitin, and several are upregulated during pressure-induced hypertrophy, including MDM2, cIAP1, c-Cbl, and E6AP [57]. During pressure overload and RGD stimulation in adult cardiomyocytes, *β*_3_ integrin regulates cIAP1 expression. Consequently, the lack of cIAP1 expression correlates with increased caspase 3 activation [20]. A similar antiapoptotic pathway has also been identified in smooth muscle cells [36]. Therefore, cardiomyocytes are able to sense the pressure/stretch stimulus from pressure overload and regulate intracellular signaling through integrin-mediated ubiquitination and elimination of targeted proteins for their survival.

c-Cbl is an E3 ligase for both RTKs and NTKs [58]. Confocal microscopic analysis of c-Cbl localization at 48 h of pressure overload shows that upregulation of c-Cbl occurs at intercalated discs [57]. In addition to RTKs, c-Cbl has been shown to ubiquitinate other receptors, including GPCRs [59] during hypertrophy. Thus, c-Cbl is linked to the endocytosis and recycling processes of cell surface receptors. Additionally, c-Cbl is also a kinase and scaffold protein in the FAC, so localization of c-Cbl to the intercalated discs during pressure overload is indicative of its role in both FAC formation and its role in ubiquitination. Ubiquitination itself may also play a role in FAC formation during pressure overload. Significantly, ubiquitination is not enhanced without FAC formation. That is, integrin stimulation with RGD in the two-dimensional cardiomyocyte model, that fails to form FAC was not accompanied by the enhanced ubiquitination whereas the three-dimensional (3D) model (detailed below) of RGD-stimulated integrin activation exhibits both FAC formation and enhanced ubiquitination [20]. These data indicate the importance of integrin activation and FAC formation for the elimination of specific target proteins via ubiquitination as part of the cell survival mechanism.

## 8. Integrin-Activating Cell Culture Model

**Figure 2:**
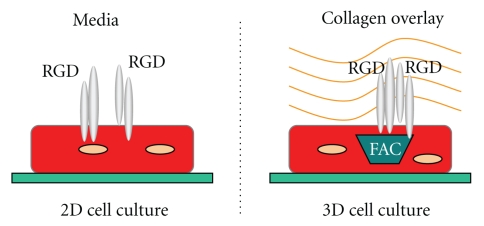
Diagram of the collagen overlay model for integrin stimulation in cardiomyocytes with RGD peptide. Cardiomyocytes are plated on laminin-coated plates in media desired for optimal experimental conditions. For integrin stimulation under two-dimensional (2D) conditions (left side of the figure), RGD peptide is added directly in the media to stimulate integrin but with no FAC. For integrin stimulation and FAC formation, (figure on the right), media are removed and replaced with the type I collagen matrix mixed with RGD peptide and allowed to polymerize. Integrins are represented by grey cylinders in the figure.

As mentioned, integrin activation and FAC formation can be recapitulated *in vitro* by embedding laminin-plated adult cardiomyocytes in a collagen matrix with the integrin-stimulating peptide RGD, referred to as a 3D model (Figure 2). This model specifically activates integrins in a similar manner as in intact tissue when the RGD motif is exposed in the extracellular matrix. When the collagen polymerizes, the embedded RGD peptide can tether to the collagen matrix to induce the integrin heterodimers on the cell surface, the most prominent being *β* **_3_** and *α*_5_ *β*_1_ [22]. To decipher signaling that is independent of the FAC formation, a 2D model can be used where RGD is added directly to media of laminin-plated cardiomyocytes. Without the stabilization provided by the polymerized collagen, the RGD is absorbed by the cardiomyocytes [32]. Devoid of the semisolid collagen matrix, RGD simply engages integrins without inducing FAC formation in the 2D model because integrins are unable to cluster and recruit focal adhesion proteins onto the cytoskeletal complex [22, 32]. Therefore, many integrin-mediated pathways requiring FAC formation for their activation are silent in this 2D model [22], such as ubiquitination [20]. On the other hand, the 3D collagen model is able to recapitulate the recruitment and activation of the *β*_3_ integrin and focal adhesion proteins, such as Src, FAK, Nck, Shc, and p130Cas, as observed in the hypertrophic animal models [22]. Importantly, introducing RGD to laminin-coated cardiomyocytes without collagen (2D model) does not cause the recruitment of these proteins to the FAC. It is also apparent that FAK is not phosphorylated during RGD treatment without FAC formation [22], indicating activation of FAK requires its recruitment to FAC. These studies lend to both pharmacological and adenoviral manipulations of cardiomyocytes, and methods have also been optimized to utilize cardiomyocytes from knockout murine models [20]. These cell culture models are important tools to decipher the intracellular signaling of integrins in cardiomyocytes.

## 9. Conclusions

Both *β*_1_ and *β*_3_ integrins are required for compensatory hypertrophic growth in response to pressure overload of the heart. Integrins interact with a complex network of proteins, which new studies continue to show are necessary for normal or hypertrophic cardiac function. The complex network of proteins transducing *β*_1_ integrin-mediated survival and growth signaling continues to grow. This includes a newly discovered interaction with ubiquitin, shown to activate PI3K/Akt signaling downstream of *β*_1_ integrin activation. Moreover, the importance of *β*_3_ integrins in ubiquitin-mediated removal of proapoptotic proteins for the survival of hypertrophying cardiomyocytes is a recent finding, and additional studies will likely decipher how this integrin subtype is tied into ubiquitination in the heart. Since integrins, PI3K/Akt, and ubiquitin signaling are each required for hypertrophic induction, continuing to study their interdependence will lead to better understanding of cardiac function during hemodynamic overload. Further study of integrin signaling may help decipher the various roles integrins play in cardiac performance and aid in the identification of potential drug targets against cardiac failure.
